# 4491 Cumulative Childhood Trauma Load Across Race in Individuals with Alcohol Use Disorder

**DOI:** 10.1017/cts.2020.119

**Published:** 2020-07-29

**Authors:** Nia Byrd, Bethany L. Stangl, Melanie L. Schwandt, Nancy Diazgranados, Vijay A. Ramchandani

**Affiliations:** 1 National Institutes of Health

## Abstract

OBJECTIVES/GOALS: Our objective was to investigate racial differences in experiencing multiple categories of childhood trauma (CT) and the differential impact on alcohol use in individuals with alcohol use disorder (AUD). We hypothesized that there would be a differential additive effect of CT categories endorsed and drinking behaviors between racial groups. METHODS/STUDY POPULATION: Participants were recruited through the NIAAA screening protocol where they completed alcohol-related assessments including a 90-day Timeline Followback (TLFB) and the Alcohol Use Disorder Identification Test (AUDIT). Structured Clinical Interviews for DSM disorders were conducted to identify participants with lifetime alcohol dependence (DSM-IV) or AUD (DSM-5) (N = 1152). Participants self-identified as Black or White completed the Childhood Trauma Questionnaire (CTQ) which assesses 5 types of CT: emotional abuse, physical abuse, sexual abuse, physical neglect, and emotional neglect, and were classified into 3 CT groups: no trauma, 1 type of trauma, and 2+ types of trauma endorsed. RESULTS/ANTICIPATED RESULTS: For Black participants (N = 583), 21.6% experienced no trauma, 21% experienced 1 type, and 57.4% experienced 2 or more types, with the most common being physical abuse and emotional neglect. For White participants (N = 569), 32.1% experienced no trauma, 20.6% experienced 1 type, and 47.3% experienced 2 or more types, with the most common being emotional neglect and emotional abuse. There were significant associations between CT groups, TLFB, and AUDIT measures. For Black participants, AUDIT-Harm and AUDIT Total were significantly different across the 3 CT groups (all p values <0.05). For White participants, Heavy Drinking Days was significantly different across the 3 CT groups (p = 0.028), with trends for AUDIT-Harm (p = 0.061) and AUDIT-Dependence (p<0.065). DISCUSSION/SIGNIFICANCE OF IMPACT: In individuals with AUD, there were significant positive associations between the number of CT categories endorsed and alcohol use across race, suggesting a cumulative effect of CT on risky alcohol use. Future work includes exploring personality and behavioral mediators of the relationship between cumulative trauma load and drinking.

